# A Spatiotemporal Deep Learning Approach for Automatic Pathological Gait Classification

**DOI:** 10.3390/s21186202

**Published:** 2021-09-16

**Authors:** Pedro Albuquerque, Tanmay Tulsidas Verlekar, Paulo Lobato Correia, Luís Ducla Soares

**Affiliations:** 1 Instituto de Telecomunicações, Instituto Superior Técnico, Universidade de Lisboa, Av. Rovisco Pais 1, 1049-001 Lisboa, Portugal; pedro.flores.albuquerque@tecnico.ulisboa.pt (P.A.); plc@lx.it.pt (P.L.C.); 2 Department of CSIS and APPCAIR, BITS Pilani, K K Birla, Goa Campus, Goa 403726, India; 3 Instituto de Telecomunicações, Instituto Universitário de Lisboa (ISCTE-IUL), Av. das Forças Armadas, 1649-026 Lisboa, Portugal; lds@lx.it.pt

**Keywords:** gait analysis, gait pathology classification, deep learning, computer vision

## Abstract

Human motion analysis provides useful information for the diagnosis and recovery assessment of people suffering from pathologies, such as those affecting the way of walking, i.e., gait. With recent developments in deep learning, state-of-the-art performance can now be achieved using a single 2D-RGB-camera-based gait analysis system, offering an objective assessment of gait-related pathologies. Such systems provide a valuable complement/alternative to the current standard practice of subjective assessment. Most 2D-RGB-camera-based gait analysis approaches rely on compact gait representations, such as the gait energy image, which summarize the characteristics of a walking sequence into one single image. However, such compact representations do not fully capture the temporal information and dependencies between successive gait movements. This limitation is addressed by proposing a spatiotemporal deep learning approach that uses a selection of key frames to represent a gait cycle. Convolutional and recurrent deep neural networks were combined, processing each gait cycle as a collection of silhouette key frames, allowing the system to learn temporal patterns among the spatial features extracted at individual time instants. Trained with gait sequences from the GAIT-IT dataset, the proposed system is able to improve gait pathology classification accuracy, outperforming state-of-the-art solutions and achieving improved generalization on cross-dataset tests.

## 1. Introduction

Gait can be defined as a sequence of limb movements that produces locomotion. Its analysis has a wide range of application in the fields of surveillance, forensics, and medicine. In the case of surveillance and forensics, gait analysis can be used to recognize individuals [1,2,3], while in the field of medicine, it can be used to monitor a patient’s recovery after a treatment, as well as help diagnose illnesses resulting from aging, injuries, or neurological disorders [4].

There are currently many systems, such as those based on instrumented facilities [5,6], wearable sensors [7,8,9], or cameras [10], that can acquire gait-related data that can be used for medical diagnoses. Of these systems, camera-based ones, such as those reported in [11,12], are becoming increasingly popular since gait video acquisition can be performed unobtrusively.

Among the camera-based approaches, the use of markers still represents the gold-standard. Solutions, such as the one reported in [13], are typically based on the application of these markers on key body parts and use multiple optical sensors to obtain kinematic features from the observed motion. However, this type of approach often relies on controlled environments and specialized personnel, as well as setup and calibration processes that can be very time-consuming and impractical in less constrained environments.

For these reasons, the work presented in this paper focuses on a markerless approach, adequate for a simple and cost-effective setup, relying on a single RGB video camera [14,15,16], as opposed to other markerless setups, which include the usage of two or more RGB video cameras [17] or cameras equipped with depth sensors [18,19,20].

Currently, approaches for gait analysis using 2D RGB video can be classified as either being appearance-based or model-based. In both cases, the objective is to try to obtain gait representations from which features describing the observed gait, both spatially and temporally, can be extracted.

Appearance-based approaches produce gait representations that do not contain any prior knowledge of human motion (e.g., binary silhouettes), but from which gait features can be extracted to analyze a person’s gait. Examples of such features include cadence and speed [14], step length, stance phase, and swing phase lengths [15], or biomechanical features such as center of gravity shifts, torso orientation, and the fraction of the cycle during which a foot is flat on the ground (i.e., flat foot fraction) [21]. A widely used gait representation for acquiring gait features is the Gait Energy Image (GEI) [22]. The GEI is obtained by averaging the aligned binary silhouettes from a complete gait cycle according to Equation (1),

(1) $$GEI\left(x,y\right)=\frac{1}{N}\sum_{i=1}^{N}B_{i}\left(x,y\right)$$

where *N* represents the number of aligned binary silhouettes, $B_{i}\left(x,y\right)$, in one or more gait cycles. The result is one single grey-scale image representing the gait information of the entire gait cycle(s). Being an average, this representation is robust against silhouette segmentation errors that may occur in isolated frames. Features such as the amount of movement or the broadness of limb movement [15] can be easily obtained from the GEI. In fact, because of this ease, the GEI has already been used as the input to a system that performs pathological gait classification based on features extracted from it [23].

On the other hand, model-based approaches attempt to fit the observations into a structural or motion model of the human body. For instance, gait sequences acquired from multiple cameras can be used to adjust a 3D human model, from which static features, such as height and leg length, and dynamic features, such as knee and ankle joint trajectories, can be extracted [24]. For instance, a representation using a 3D skeleton, obtained from an RGB + depth camera, was considered in [18] to detect abnormal gait. It uses a spatiotemporal feature, the joint motion history, which represents the motion of skeleton joints over a sequence. In [25], a skeleton was fit to the observed gait images to create a compact gait representation called the Skeleton Energy Image (SEI). The skeleton model was fit by extracting the 2D coordinates of several key human body parts using OpenPose [26]. The SEI was then obtained similarly to Equation (1), but now using the skeletons computed for every frame instead of silhouettes. This approach reported an improvement in pathological gait classification results compared to using the GEI, as it decouples motion information from the appearance characteristics of a subject.

Independent of the approach followed (appearance-based or model-based), with the aim of improving performance, most recent markerless approaches are now based on deep learning strategies, although they are still mostly based on well-known gait representations, such as the GEI. They use Convolutional Neural Networks (CNNs), as these architectures are able to learn visual features with a high level of abstraction. For instance, in [27], pathological gait was analyzed using the VGG-19 architecture [28], pretrained with a subset of the ImageNet database [29]. Transfer learning was used to repurpose the model for gait classification using the GEIs computed from the INIT gait dataset sequences [15]. Principal Component Analysis (PCA) was then applied to the output of the convolutional layers for dimensionality reduction, and classification used Linear Discriminant Analysis (LDA). The system was evaluated on the DAI [16] and DAI 2 [23] datasets, which contain normal and impaired gait and normal and four different gait-related pathology sequences, respectively. The work reported in [25] also used the VGG-19 architecture for pathological gait classification. The original model was fine-tuned using the GEI and SEI representations computed from the GAIT-IST dataset [30], containing ten subjects performing their normal gait and simulating four different gait-related pathologies.

Given that CNNs are mostly suited for extracting spatial features from images, applying them directly to gait representations such as the GEI or SEI, where the temporal dimension has already been collapsed into a single image, may not be the most effective way to analyze gait data. After all, temporal features can also play a significant role in gait pathology classification. Recurrent Neural Networks (RNNs) are neural networks that learn dependencies between inputs in a time series, thus being suitable for extracting temporal features from a video sequence. An individual’s movement at a given time instant, during locomotion, is significantly related to the movement in the previous instant and influences the movement that will occur next. This was explored in [31], where a bidirectional Long Short-Term Memory (LSTM) network was used to classify gait as being either normal or pathological. A depth-sensing camera allowed estimating the orientation of body joints, used to estimate the lower limb flexion angles, which were the input to the LSTM-based classification system. However, its range of operation in terms of depth was quite limited, and the classification performed was binary. Furthermore, depth sensing cameras are not always readily available, unlike RGB cameras, which are present even on inexpensive cell phones.

Combined CNN-LSTM networks have shown promising results for classifying different human activities using a simple RGB camera [32]. Thus, in this paper, it is hypothesized that a more complex classification of gait-related pathologies was possible with a combination of CNN and LSTM, using a significantly simpler RGB camera. In order to prove this, a deep learning framework that focuses both on spatial and temporal features is proposed. A new suitable gait representation is also defined to be used as the input. This way, in the context of automatic pathological gait classification using video captured from a single RGB camera, this paper makes the following two main contributions:

- Representation by a selection of key frames—A complete gait cycle is represented by a set of key silhouette frames, obtained by the proposed frame selection module. These key frames are selected taking as reference the functional phases expected to be present in normal gait, according to clinical gait literature [33]: initial contact, mid stance, terminal stance, preswing, initial swing, midswing, and terminal swing. This representation provides more information than the compact ones often considered by state-of-the-art solutions, such as the GEI. In particular, it allows a deeper analysis of the temporal dependencies between consecutive moments during human locomotion;
- Spatiotemporal deep learning framework for classification—The proposed pathological gait classification framework combines convolutional and recurrent neural networks to extract both spatial and temporal features from the set of key frames used to represent gait. A CNN is used to extract spatial features from each key frame, at individual time instants. Then, the adopted RNN, an LSTM, is used to learn temporal patterns of the spatial features along the gait cycle. Gait can then be classified into one of the five gait classes considered: diplegic, hemiplegic, neuropathic, Parkinsonian, or healthy/normal gait.

The proposed system was evaluated using the GAIT-IST [25] and GAIT-IT [34] pathological gait datasets, with a total of ten and twenty-one different healthy subjects, respectively, performing their normal gait and simulating each pathology with two levels of severity. For every gait sequence, both datasets provide binary silhouettes, binary skeletons, and GEI and SEI representations obtained from a sagittal view. The silhouettes were obtained using background subtraction techniques such as Gaussian mixture models and chroma-keying segmentation [25,34]. These techniques generate a model for the background using sample frames, thus making them robust to background clutter and illumination changes. A silhouette was generated from the difference between the background model and an input frame.

## 2. Materials and Methods: Proposed Gait Analysis System

The state-of-the-art pathology classification systems rely on spatial features obtained from compact gait representations, such as the GEI [22]. These representations are robust and compact, including spatial and motion-related information in a single image. The best reported results when using such representations were obtained using CNNs, such as VGG-19 [25,27].

However, as discussed above, the GEI cannot accurately seize all the temporal information present in a video sequence, notably the relationships between the movements captured at each time instant. If captured at the right time instants, these relationships can be extremely valuable to characterize gait because, as shown in clinical research, gait patterns can be observed and determined through a sequence of events characterized by their functional purpose [33].

To explore the temporal information in a video sequence, the proposed approach combines the spatial and temporal feature extraction abilities of convolutional and recurrent neural networks, respectively. It processes gait cycles as a collection of binary silhouettes present in frames captured at specific time instants. The proposed framework thus learns the temporal patterns associated with the inherent order and dependencies among the spatial features obtained at different moments in a gait cycle. The recurrent neural network architecture considered to process the temporal information was the LSTM network [35], since it addresses the limitation of RNNs to store long-term dependencies and it regulates the information that flows through the network.

**Figure 1:**
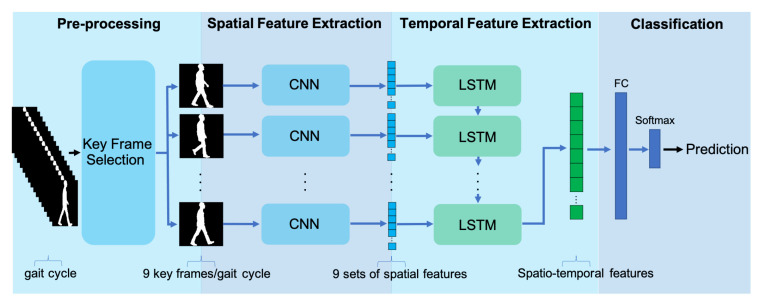
Architecture of the proposed spatiotemporal deep learning framework for pathological gait classification. In the figure, the same CNN is repeated multiple times for illustrative purposes.

The proposed gait analysis framework performs pathological gait classification from a sequence of frames that constitute a gait cycle. In the following implementation, the binary silhouettes from the GAIT-IT pathological gait dataset were considered as the input data. The architecture of the proposed spatiotemporal deep learning framework for pathological gait classification is illustrated in Figure 1. It consists of 4 main modules:

- Key frame selection—This module selects 9 key frames to represent 1 gait cycle, with each key frame containing a binary silhouette;
- Spatial feature extraction—This module uses a pretrained CNN, which is used to process each of the 9 selected key frames individually, thus obtaining 9 sets of 2D spatial features, each flattened into a 1D vector;
- Temporal feature extraction—This module processes the sequence of spatial features extracted from the 9 key frames representing one gait cycle, using a bidirectional LSTM network, to obtain the desired spatiotemporal features;
- Classification—The final module uses the features extracted in the previous module to reach a classification decision, by using a dense neural network of fully connected layers.

### 2.1. Key Frame Selection

The key frame selection module selects 9 key frames in each gait cycle to represent the locomotion progression of the subject.

**Figure 2:**
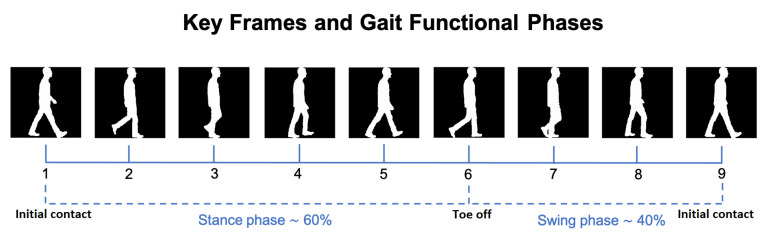
Example of the silhouettes in the 9 key frames representing a normal walking gait cycle, along with the 8 gait phases in between consecutive key frames.

According to clinical research [33], a complete gait cycle from a healthy subject consists of eight distinct functional phases, as represented in Figure 2. By analyzing a subject’s gait during those phases, the deviation from healthy gait can be identified as an impairment, possibly associated with some gait pathology. This served as the motivation for the proposed frame selection procedure, where 9 key silhouette frames were selected from those available in each gait cycle, corresponding to the those 8 phases.

The first of the 9 key frames marks the beginning of the gait cycle (and also the end of the previous one), while the last of the 9 key frames marks the end of the gait cycle. The other 7 intermediate key frames represent consecutive phases.

**Figure 3:**
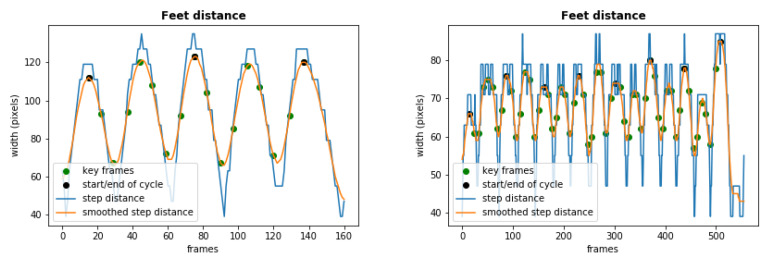
Feet distance plot along the frames highlighting the extracted 9 key frames in a normal gait sequence (**left**) and a Parkinsonian gait sequence (**right**).

In order to obtain these key frames, the first step is to identify each gait cycle in a video sequence. This can be achieved by plotting the distance between both feet along a sequence (see Figure 3), with each gait cycle corresponding to the frames within three consecutive maximum distance peaks. The first and last key frames (i.e., #1 and #9) are the frames where the first and third of those three peaks occur. The next step is to obtain the other 7 key frames to be used for gait analysis. The transition between the stance and swing phases occurs at nearly 60% of a complete cycle in healthy gait [33]. This corresponds to the transition between the fifth and sixth phases, and Key Frame #6 is obtained by marking this transition. This key frame acts as a reference for the rest of the selection process. The stance phase is divided into 5 equal portions, allowing obtaining the 4 key frames numbered #2, #3, #4, and #5. The same is done for the swing phase of the gait cycle, which is divided into three equal portions, identifying the two remaining key frames (i.e., #7 and #8).

**Figure 4:**
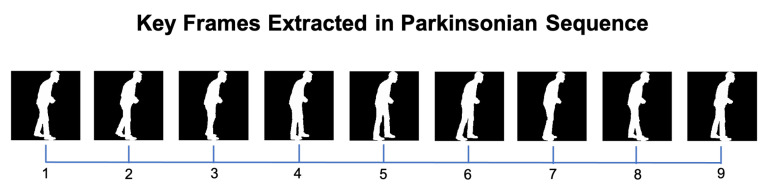
Example of silhouettes in the 9 key frames representing a Parkinsonian gait cycle, extracted according to the proposed key frame selection module.

An example of the key frames computed for a subject’s normal gait can be seen in Figure 2. For comparison, also the key frames representing a gait cycle from a simulated Parkinsonian gait are illustrated in Figure 4, portraying the differences in the observed motion at the same relative moments of the gait cycle. The plots in Figure 3 represent the distance between both feet along a gait cycle, highlighting the 9 selected key frames. The left and right plots correspond to the normal and Parkinsonian gait cycles illustrated in Figure 2 and Figure 4, respectively.

### 2.2. Spatial Feature Extraction

In the first stage of feature extraction, the objective is to obtain a set of compact representations from each of the 9 key frames.

To achieve this, the pretrained convolutional base of the VGG-16 network [28] was considered for fine-tuning. The convolutional base of VGG-16 includes 13 convolutional layers (organized into 5 convolutional blocks) before the last 3 fully connected layers. The fine-tuned VGG architecture was chosen because it has demonstrated its effectiveness in performing classification of pathologies using small datasets, without overfitting to the data [25,27,36,37,38]. The convolutional base of the VGG-16 network was used to process each of the 9 key silhouette frames independently, yielding a set of 2D spatial features for each. The 9 sets of spatial features were then flattened into 9 1D vectors and used as the input by the LSTM network in the temporal feature extraction module.

To fine-tune the VGG-16 convolutional base, a dense network was stacked on top of the convolutional layers to perform the classification. The dense network consisted of two fully connected layers of 4096 units and a softmax layer with 5 units, corresponding to the desired output classes, representing the 5 different gait types in the GAIT-IT dataset. The fine-tuning process only retrained the last 3 convolutional blocks of the 5 available in the VGG-16 network; the first 2 convolutional blocks with 2 convolutional layers each were frozen, allowing the parameters of the following layers to be updated. After fine-tuning, the last two layers of the dense network classifier were removed (i.e., the second fully connected layer with 4096 units and the softmax layer), resulting in a flattened 1D feature vector of size 4096 as the output.

### 2.3. Temporal Feature Extraction

The advancing movements that produce locomotion can be successfully analyzed using a recurrent neural network. The RNN captures the relevant motion patterns from the relationship between subsequent gait key frames, obtained at specific moments in a gait cycle. For a more complete analysis, a bidirectional recurrent neural network [39] can be employed, consisting of two recurrent networks processing the same set of input frames simultaneously, with one handling the input sequence in its natural order while the other one processes them in reverse order. This is a widely used approach in contexts where the entire input sequence is available at prediction time [31,40,41]. A bidirectional RNN can thus explore additional information from that same sequence.

**Figure 5:**
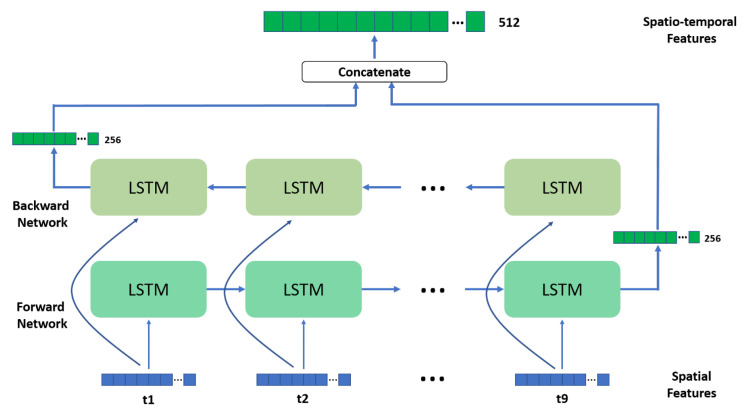
Illustration of the temporal feature extraction module, using a BiLSTM architecture.

The temporal feature extraction module of the proposed framework considers a Bidirectional LSTM (BiLSTM), assuming that a complete gait cycle is available as the input. The sequence of flattened spatial features obtained from the CNNs, for the 9 key frames, is processed by the BiLSTM network. Each of the two LSTM networks consists of 9 LSTM cells, each accepting 1 spatial feature vector from the input sequence, as illustrated in Figure 5. The LSTM architecture shares a cell state to facilitate the flow of information through the entire chain, keeping a long-term memory. Each LSTM cell also includes other units (i.e., input gate, forget gate, and output gate) that regulate how to update the cell state and the cell output (hidden state), at each time step [35]. The implemented BiLSTM network yields a 1D vector of spatiotemporal features, of size 512, composed of two concatenated outputs, each of size 256, obtained from the last LSTM cell from each direction. The output from the BiLSTM is directly used as an input by the classification module.

### 2.4. Classification

The classification module consists of a neural network composed of 2 fully connected layers with a dropout of 0.5 between them. Dropout was used as a regularizer to limit overfitting. During each epoch, the inputs or outputs of a given layer were randomly removed, along with the incoming and outgoing connections [42]. This allows each update to have a different perspective of the configured layer.

The first fully connected layer has a dimension of 512 units, to match the length of the BiLSTM module’s output. The second fully connected layer has 5 units, with a softmax activation function, which outputs the probabilities of the input belonging to each of the considered gait type classes.

The network was trained using categorical cross-entropy as the loss function and the Adam optimizer [43] with Nesterov momentum variation. The learning rate was set to 0.0001. The Adam optimizer has been widely adopted for deep learning problems and demonstrated to compare favorably to other stochastic optimizers, while being computationally efficient [43]. As illustrated in [44], the implementation of Nesterov’s accelerated gradient algorithm to modify Adam’s momentum can further improve the quality and convergence speed of the learned models.

## 3. Results

The proposed framework was evaluated on the GAIT-IT and GAIT-IST datasets of simulated pathological gait (by healthy individuals). For both datasets, sequences of binary silhouettes are available, together with ground truth classification labels, classifying them as healthy/normal gait or as one of four different pathological gaits considered: diplegic, hemiplegic, neuropathic, and Parkinsonian. The datasets consisted of subjects walking twice from left to right and from right to left, observed from a sagittal perspective. Furthermore, each pathological gait was simulated with two levels of severity, varying the degree to which the observed locomotion was affected by each impairment.

The acquisition of the gait data for GAIT-IT was performed on two different days, with prior training offered through videos, live demos, and practice sessions. To allow studying intrasubject variations, two subjects repeated the acquisition of their gait sequences on both days. This means that GAIT-IT featured 21 different subjects with 23 different subject performances of each gait type.

Two evaluation scenarios were considered: (i) using cross-validation, on a single dataset; (ii) performing a cross-dataset evaluation, where the proposed spatiotemporal deep learning model was trained using one dataset and then tested using a different dataset.

### 3.1. Cross-Validation Results

The first evaluation scenario used only the GAIT-IT dataset for training and testing. In this case, a 10-fold cross-validation approach was adopted, considering all subject gait sequences except the two subject repetitions. The test set for each fold included three subjects and is defined as $V_{k}=\left\{S_{i},S_{i+1},S_{i+2}\right\}$, where k=1,2,…,10 is the fold iteration, i=2×k−1, and $S_{i}$ represents all sequences from one of the total of twenty-one subjects, following the numbered labels used for each subject in the dataset. The other 18 subjects composed the corresponding training set. Thus, with the considered 10-fold cross-validation approach, all available subjects were included at least once in the test set, while no subjects were ever simultaneously present in the training and test sets, thus minimizing bias in the training set. Given the reduced size of the available GAIT-IT dataset, no subjects were used for the tuning and validation of the hyperparameters; the values of the original network were used instead (e.g., a learning rate of 0.0001).

Results are presented in Table 1, in the form of a confusion matrix, showing the classification accuracy for each class as the average of the 10 folds (with the standard deviation shown between parentheses). The confusion matrix provides valuable insights regarding the system’s effectiveness in detecting a given pathology. It also identifies the types of impairments that are more difficult to classify or distinguish. The overall accuracy of the system was computed as the mean value of the matrix diagonal, which consisted of taking the average of the correct classification rate for each class.

The proposed system was also compared to the state-of-the-art VGG-19 [28] Convolutional Neural Network (CNN) model, pretrained on ImageNet [29], and fine-tuned using pathological gait datasets, as described in [25]. For each test, the VGG-19 approach was evaluated using the GEI and SEI gait representations as the input, and their respective mean classification accuracies are reported in Table 2, along with that achieved by the proposed spatiotemporal deep learning gait classification framework.

### 3.2. Cross-Dataset Results

The second, more challenging, evaluation scenario consisted of a cross-dataset evaluation, to understand the generalization capability of the proposed system. For this scenario, the spatiotemporal deep learning pathological gait classification framework was trained using all twenty-three subject gait sequences (i.e., including the repetitions from two of the twenty-one subjects, as previously mentioned) from GAIT-IT. Then, the model was tested on all 10 subjects from the GAIT-IST dataset. It should be noted that the subjects recorded in the GAIT-IT dataset were different from the GAIT-IST dataset. Furthermore, the recording for the GAIT-IST dataset was carried out using a cell phone camera in a relatively noisy environment. Even the distance between the camera and the subjects was not consistent across the datasets. This led to significantly different silhouettes in the two datasets, thus making the cross-dataset evaluation challenging. The corresponding results are presented in Table 3, again comparing against the solutions described in [25], taking the GEIs or SEIs as the input.

## 4. Discussion

By analyzing the results obtained in the confusion matrix displayed in Table 1, the performance of the proposed system can be assessed with respect to each pathology. A first observation regards the classification accuracy of normal gait sequences. It represents the highest classification accuracy (99%) among all considered gait types. On the other side, the classification of diplegic gait achieved the lowest accuracy (92%). The confusion matrix shows that diplegic gait was sometimes mislabeled as hemiplegic or Parkinsonian, in 8% of the analyzed gait sequences, which can be argued to be the most challenging differentiation for the system. In fact, diplegic and hemiplegic simulations have similarities when observed from the sagittal view, specifically when the subject is simulating the hemiplegic pathology with the affected side facing the camera. Furthermore, both diplegic and Parkinsonian gaits are characterized by a stooped posture and small steps. Hemiplegic gait had an average classification accuracy of 96%. In the case of the classification error, it was mostly mistaken for the neuropathic gait. Neuropathic gait achieved an average accuracy of 98%. Parkinsonian gait also had an accuracy of 98% and was mislabeled as diplegic gait in approximately 1% of the predictions.

The overall classification accuracies displayed in Table 2 and Table 3 suggest that the use of the proposed spatiotemporal deep learning gait classification framework, relying on the combination of a CNN and a bidirectional LSTM, provides a significant advantage. While both tested systems employed a fine-tuned VGG network as the convolutional base, the proposed spatiotemporal deep learning framework showed an improvement in performance in both scenarios. Cross-validation on GAIT-IT using the proposed spatiotemporal deep learning framework achieved a classification accuracy of 96.5%, representing an increase of 2.5% and 2.9% when compared with the performance of the VGG-19 approach [25] using GEI and SEI, respectively. Results for the cross-dataset evaluation scenario showed the proposed spatiotemporal deep learning framework reaching a classification accuracy of 91.4%, corresponding to a significant improvement, of 5.0% and 6.3%, in the overall performance when compared to state-of-the-art results on the GEI and SEI, respectively. Given the presence of segmentation errors in binary silhouettes available in GAIT-IST, the results suggest that the proposed system has a good generalization capability, while also being robust to noise in the binary silhouettes provided as input to the system.

## 5. Conclusions

In this paper, a video-based spatiotemporal deep learning framework for pathological gait classification was proposed and evaluated. It combines both convolutional and recurrent deep neural networks, for the extraction of spatiotemporal features from gait sequences represented by a selection of binary silhouette key frames. The combination of a fined-tuned VGG-16 CNN with a bidirectional LSTM resulted in a solution able to surpass state-of-the-art results in cross-validation tests on the GAIT-IT dataset. It also achieved a very interesting overall accuracy of 91.4% in a cross-dataset evaluation scenario, using the GAIT-IT dataset for training and the GAIT-IST dataset for testing. The cross-dataset scenario hinted that the proposed spatiotemporal deep learning gait classification framework has a good generalization ability. Moreover, the cross-dataset evaluation also gave an indication that the proposed framework is robust to noisy input silhouettes, performing well even in the presence of segmentation errors on the binary silhouettes available from the GAIT-IST dataset.

For future work, the use of a Convolutional LSTM (ConvLSTM) [45] can be considered as an alternative to the LSTM network for the temporal feature extraction module described in Section 2.3. The ConvLSTM extends the LSTM model with convolutional operations to replace internal matrix multiplications. This allows the ConvLSTM cells to maintain the 2D input dimension as the information flows through the network, which makes this architecture well-suited for capturing spatiotemporal correlations.

Furthermore, since the GAIT-IT dataset includes gait sequences recorded with two synchronized cameras, capturing both sagittal and frontal points of view, a solution exploring both types of inputs could significantly increase the amount of information available for pathological gait classification, notably to help distinguish some types of gait impairments. Furthermore, alternative LSTM architectures that allow the simultaneous processing of multiple synchronous sequences could also be considered, for instance using a solution similar to the one proposed in the context of facial recognition [46]. The proposed spatiotemporal deep learning gait classification framework could be combined with that architecture to create a system able to simultaneously explore frontal and sagittal gait sequences.

## Figures and Tables

**Table 1:** Cross-validation results of the proposed spatiotemporal deep learning gait classification framework: confusion matrix with classification accuracies (and the standard deviation shown between parentheses), in %, on the GAIT-IT dataset; the overall average accuracy was 96.5%.

Predicted Class						
		Diplegic	Hemiplegic	Neuropathic	Normal	Parkinson
**True Class**	Diplegic	92 (10)	4	0	0	4
	Hemiplegic	1	96 (2)	3	0	0
	Neuropathic	0	2	98 (4)	0	0
	Normal	0	0	1	99 (3)	0
	Parkinsonian	1	0	0	0	98 (3)

**Table 2:** Cross-validation average accuracy results (%) on the GAIT-IT dataset: comparison against a state-of-the-art solution using a VGG-19 CNN with the GEI or SEI gait representations.

Pathological Gait Classification System	Input Type	Accuracy
VGG-19 [25]	GEI	94.0
VGG-19 [25]	SEI	93.6
VGG-16-BiLSTM	Binary Silhouettes	96.5

**Table 3:** Cross-database average accuracy results (%) of the proposed spatiotemporal deep learning gait classification framework: using GAIT-IT for training and GAIT-IST for testing; results are compared against a state-of-the-art solution using a VGG-19 CNN with the GEI or SEI gait representations.

Pathological Gait Classification System	Input Type	Accuracy
VGG-19 [25]	GEI	86.4
VGG-19 [25]	SEI	85.1
VGG-16-BiLSTM	Binary Silhouettes	91.4
